# The association between *Acinetobacter baumannii* infections and the COVID-19 pandemic in an intensive care unit

**DOI:** 10.1038/s41598-022-25493-8

**Published:** 2022-12-02

**Authors:** Jale Boral, Zeliha Genç, Fatihan Pınarlık, Güz Ekinci, Mert A. Kuskucu, Pelin İrkören, Mahir Kapmaz, Süda Tekin, Nahit Çakar, Evren Şentürk, Fatma Yurdakul, Bilge Dikenelli, Fusun Can, Onder Ergonul

**Affiliations:** 1 Graduate School of Health Sciences, Koç University, Istanbul, Turkey; 2 Koç University İşBank Center for Infectious Diseases, Koç University Hospital, Maltepe Mahallesi, Davutpaşa Caddesi, No:4 Topkapı, 34010 Istanbul, Turkey; 3 Infectious Diseases and Clinical Microbiology, Koç University Hospital, Istanbul, Turkey; 4 Department of Medical Microbiology, Cerrahpasa School of Medicine, Istanbul, Turkey; 5 Department of Infectious Diseases and Clinical Microbiology, School of Medicine, Koç University, Istanbul, Turkey; 6 Department of Anesthesiology and Reanimation, Koç University School of Medicine, Istanbul, Turkey; 7 Clinical Microbiology Laboratory, Koç University Hospital, Istanbul, Turkey

**Keywords:** Clinical microbiology, Microbiology, Health care

## Abstract

We aimed to describe the increased rate of *Acinetobacter baumannii* infections during the COVID-19 pandemic and define its significance within the last five years. This study was performed in a tertiary hospital with 280 beds and included all patients infected with *A. baumannii* in the intensive care unit between January 1, 2018, and June 30, 2022. *A. baumannii*-infected patients in the intensive care unit 27 months before the pandemic and 27 months during the pandemic were included. Pulsed-field gel electrophoresis was performed to assess clonal relatedness. The infection control measures were specified based on the findings and targeted elimination. In total, 5718 patients were admitted to the intensive care unit from January 1st, 2018, to June 30th, 2022. *A. baumannii* infection was detected in 81 patients. Compared to the pre-pandemic era, the rate of *A. baumannii* infection during the pandemic was 1.90 times higher (OR: 1.90, 95% CI: [1.197, 3.033]). Clonality assessment of multidrug-resistant *A. baumannii* samples revealed eight clusters with one main cluster comprising 14/27 isolates between 2021 and 2022. The case fatality rate of the pre-pandemic and pandemic era was not different statistically (83.33% vs. 81.48%, p = 0.835). Univariate analysis revealed the association of mechanical ventilation (p = 0.002) and bacterial growth in tracheal aspirate (p = 0.001) with fatality. During the COVID-19 pandemic, potential deficits in infection control measures may lead to persistent nosocomial outbreaks. In this study, the introduction of enhanced and customized infection control measures has resulted in the containment of an *A. baumannii* outbreak.

## Introduction

*Acinetobacter baumannii* is an aerobic, non-motile, gram-negative bacteria, one of the leading causes of nosocomial infections ^1^. *A. baumannii* is commonly asserted to cause infection in immunocompromised patients with an increased tendency of infection as a result of exposure to broad-spectrum antibiotics and disruption of anatomic barriers with the use of ventilators, central lines, and urinary catheters where there is a high rate of intervention with patients^2^. Although its ability to anchor with cells and mucosal cells is low compared to other microorganisms such as a *Pseudomonas aeruginosa*, *Neisseria meningitidis*, *Campylobacter* spp., *Yersinia enterocolitica* and *Helicobacter pylori*, its ability to survive at dry surfaces and high temperatures for longer makes it more persistent and spreadable in healthcare environments^3,4^.

Carbapenem-resistant *A. baumannii* has been listed as “Priority 1: Critical” bacteria for resistance, surveillance, and discovery of new antibiotics by WHO in 2017^5^ and Turkey is one the countries with the highest reported rate (95%) of carbapenem-resistant *A. baumannii*^6^. As of 2018, colistin resistance of *A. baumannii* in Turkey was reported to be seven percent^6^, this diminishes the likelihood of pandrug-resistant (PDR) *A. baumannii* isolates, however, extensively drug-resistant (XDR) *A. baumannii* is reported to be an emerging problem^7^.

The lack of effective antimicrobial treatment against carbapenem-resistant *A. baumannii* signifies the need for successful compliance with infection control practices.

Several studies reported an increased risk of carbapenem-resistant *A. baumannii* infections in patients with an increased risk of mortality due to COVID-19 infections^8–10^. Increased incidences of *A. baumannii* infections during the COVID-19 pandemic were linked to various reasons such as prolonged hospital stay, invasive and non-invasive mechanical ventilation, and immunosuppression^11–14^.

Despite antibiotic resistance being the primary driver of clinical outcome in *A. baumannii* infections, the virulence properties such as capsular lipopolysaccharide presence and biofilm formation, effectively trigger lipopolysaccharide (LPS)–Toll-like receptor 4 (TLR4)-mediated sepsis by reaching high bacterial density in a short time span^2^. Given the prevalence of antibiotic resistance and successful virulence mechanisms of *A. baumannii,* co-occurrence of the COVID-19 and *A. baumannii* should be studied in terms of association and clinical outcome as the detrimental prognosis of this co-infection may be a rapid-onset^15^.

Due to an increase in *A. baumannii* infections in 2021 at a COVID-19 ICU, we aimed to describe the association between COVID-19 and *A. baumannii* co-infections by comparing infection rates with pre-COVID-19 pandemic data. This abrupt increase also required an assessment of isolates on a molecular basis by a genotyping method such as Multilocus Sequence Typing (MLST), Whole Genome Sequencing (WGS), or Pulsed Field Gel Electrophoresis (PFGE) to determine the clonal relatedness^16,17^. In this study, we additionally present suggestions for the containment and elimination of *A. baumannii* infections during the COVID-19 pandemic era regarding international infection control measures.

## Materials and methods

### Study population, diagnostic criteria, and sample collection

The study was conducted at Koç University Hospital, Istanbul, Turkey. All the patients infected with *A. baumannii* in the ICU were included, covering 27 months for both the pre-pandemic (January 2018–March 2020) and the pandemic (April 2020–June 2022) era. Clinical findings for sepsis^18^, pneumonia^19^, and/or urinary tract infection^20^ and isolation of *A. baumannii* from blood, urine, and/or tracheal aspirate were required for the diagnosis of infection. Bacterial identifications were done using MALDI-TOF VITEK® MS (BioMerieux, March L’Etoile, France).

### Antibiotic susceptibility testing

Antimicrobial susceptibility testing was performed for piperacillin-tazobactam, ceftazidime, imipenem, meropenem, amikacin, gentamicin, ciprofloxacin, tigecycline, and colistin by using the Vitek2 automated system (BioMerieux, March L’Etoile, France). The MIC breakpoints were evaluated according to the EUCAST criteria^21^. *A. baumannii* isolates testing resistant to at least one antibiotic of more than three antibiotic classes were identified as multidrug-resistant (MDR) isolates. The presence of carbapenemase was checked using the RAPIDEC® Carba NP kit (BioMerieux, March L’Etoile, France). Confirmation of amikacin susceptibility was verified using the disk diffusion method with 30 μg of amikacin-containing discs.

### Pulsed-field gel electrophoresis and cluster analysis

Pulsed-field gel electrophoresis (PFGE) method was done to assess the genetic clonality of 27 MDR *A. baumannii* isolates collected from the patients admitted to the ICU between January 2021 and February 2022 during the COVID-19 pandemic. The salmonella standard protocol of CDC PulseNet was used with minor modifications^22^. Bacterial samples cultured overnight were adjusted to the cell concentration of 0.45 absorbance at 590 nm (2.00 McFarland Standard) and suspended in the cell suspension buffer (100 mM Tris (pH 8.0), 10 mM EDTA). Low melting 1% SeaKem agarose was used for the formation of the plugs. Genomic DNA was lysed overnight at 55 °C in a lysis solution (50 mM Tris, 50 mM EDTA, proteinase K (20 mg/ml), pH 8.0). ApaI (50U per isolate) was used as the restriction enzyme and incubation was done at 37 °C for 4 h. Lambda Ladder PFG Marker (NEB, US) was used as the molecular size marker and 1% SeaKem agarose was used for gel preparation. Run conditions were set as, temperature 14 °C; voltage 6 V/cm; switch angle, 120°; switch ramp 7–20 s with the run duration of 18 h on Chef Mapper II (Chef Mapper, Bio-Rad Laboratories, Hercules, CA, USA). Ethidium bromide was used for gel staining and visualization of the results was done using ChemiDoc™ XRS + System with Image Lab™ Software (BIO-RAD, USA).

*Acinetobacter baumannii* ATCC 17978 was used for the normalization of bands on different gels and the oldest isolate was used as the reference strain, timewise. Results were analyzed using Bionumerics 7.6 software (Applied Maths NV, St-Martens-Latem Belgium) using the Dice correlation coefficient. The dendrogram illustrating the clonal relatedness of isolates was plotted using the Unweighted Pairgroup Method with Arithmetic Averages (UPGMA) with a position tolerance value of 1%. Clonality scores of 85% and above were accepted as clonal isolates^23,24^.

### Infection control measures before the outbreak

Education regarding cleaning, hand hygiene, disinfection, basic microbiology, and infection control was delivered as a part of the introduction program to all healthcare staff right after recruitment. Healthcare staff was monitored and assessed every month regularly for their compliance with infection control measures. Feedback on hand hygiene was delivered monthly, based on the monitoring results generated by anonymous observers. The hand hygiene score was calculated, and the goal was set to be greater than 90%. Education on appropriate hand hygiene was given by the infection control team after receiving feedback. The importance of aseptic techniques during aspiration was also emphasized. The surfaces of aspiration jars were wiped using an alcohol-based disinfectant. Right after the initiation of the pandemic, double gloves were in use. Cleaning solutions used for surfaces and appliances were peracetic acid solution (0.2%) and chloride solution (0.1%).

### Infection control measures during the outbreak

*Acinetobacter baumannii* infection elimination program was created by the infection control team regarding infection prevention and control (IPC) measures^25^. This program consisted of extended measures on appropriate personal protective equipment (PPE) use, environmental screening, hand hygiene, isolation precautions, cleaning, and disinfection with the participation of nurses, doctors, and janitorial staff. Infection control measures before the *A. baumannii* outbreak and additional measures during the outbreak were compared in Table 1.

**Table 1:** Infection control measures before and after *A. baumannii* outbreak.

	Before *A. baumannii* outbreak	During *A. baumannii* outbreak
**Training of healthcare workers**		
PPE training	After recruitment	On daily basis
Preparation of cleaning solutions	After recruitment	On daily basis
Hand hygiene score	70%	97%
Glove usage	Double glove	Single glove
**Cleaning procedures**		
Types of cleaning solutions	Peracetic acid solution (2.0%) or chloride solution (0.1%)	Only chloride solution (0.1%)
Aspiration jars	Cleaned with surface wiping	Soaked in chloride solution
Cleaning routine	Single cleaning	Double cleaning
**Ventilator related precautions**		
Appropriate ventilator cleaning procedures	Standard cleaning procedures for ventilators	Separate procedures for each device
Ventilator cleaning	Ventilator cleaning	Ventilator disinfection
Transport ventilator filters	Inhalation port filter was changed	Filters for both inhalation and exhalation ports were changed
**Environmental measures**		
Environmental screening	None	*A. baumannii* infected rooms
Clonality surveillance	None	PFGE
Isolation of COVID-19 patients in rooms 1–8	Yes	Yes

Monitoring across all shifts was conducted daily under the supervision of the lead nurse of the infection control team. COVID-19 and *A. baumannii* infection patients were followed on contact, droplet, and airborne precautions. Non-disposable equipment was cleaned using 1000 ppm (0.1%) chlorine solution daily and for each visit. The use of double gloves was terminated as it has led to the continuation of using the inner glove after disposing of the outer glove. Aspiration jars were disinfected by soaking in 0.1% hypochlorite solution instead of wiping the surface of the jars with a chlorine solution (0.1%). Disposal of PPE was done after each examination and bedside visit. All equipment was disinfected before the removal from the room. Healthcare workers have received training on appropriate donning and doffing of PPE (gowns, gloves, goggles, or face shields). Hand hygiene monitoring has been implemented weekly instead of monthly.

The environmental screening was performed by infection control nurses in the patient rooms where *A. baumannii* was isolated after cleaning. Screening consisted of door handles, bedsides, IV pumps, aspiration jars, drawers, the surface of alcohol-based disinfectant bottles, monitors, ventilators, and stethoscopes per each room. Each room was blindly cleaned twice by two janitorial staff after each patient. Extended cleaning and disinfection training was given to all janitorial staff as practice (chlorine solution preparation, cleaning procedures, use of a single cleaning cloth). Clonality surveillance of *A. baumannii* isolates was done using the PFGE method.

Eventually, COVID-19 and *A. baumannii* infection patients were mainly isolated in rooms 1–8 which is a separate area within the ICU. The patients were taken to the other corridor with room numbers 9–16 only when rooms 1–8 were fully occupied.

### Statistical analysis

Descriptive statistics of continuous variables were performed with medians and interquartile ranges. For categorical values frequencies were compared. The chi-square test and Mann–Whitney U Test were used to analyze the relationship between binary variables and continuous variables respectively. All the analyses were conducted via Stata 17.0 (StataCorp LLC, Texas USA) and a p-value less than 0.05 was considered statistically significant.

### Ethics approval

All methods were carried out in accordance with relevant guidelines and regulations after receiving ethics approval. This study was approved by the Koç University Institutional Review Board (No: 2022.192.IRB1.069). Informed Consent was obtained from all patients included in the study.

## Results

A total of 5718 patients were admitted to ICU from January 1st, 2018, to June 30th, 2022. Among 5718 patients, *A. baumannii* infection was detected in 81. The rate of *A. baumannii* infection during the pandemic was 1.90 times (OR: 1.90, 95% CI: [1.197, 3.033]) higher compared to the pre-pandemic era.

The demographic and clinical characteristics of the patients and univariate analysis for the 30-day fatality were described in Table 2. The trachea was the major infection site (69/81), followed by bloodstream infections (24/81) and urinary infections (1/81). In univariate analysis, a history of mechanical ventilation (p = 0.002) and bacterial growth in tracheal aspirate (p = 0.001) were associated with fatality. The history of mechanical ventilation was higher (98.41%) for fatal patients compared to surviving patients (78.57%). Similarly, the percentage of bacterial growth from tracheal aspirates was 91.04% in fatal cases while it was 57.14% in surviving cases.

**Table 2:** Demographic and clinical characteristics of patients with *A. baumannii* infection.

	Total (%) N = 81	Fatal (%) N = 67	Survived (%) N = 14	*p-value*
**Period**				
Pre-Pandemic, 27 Months (January 2018–March 2020)	27 (33.33)	22 (32.84)	5 (35.71)	0.835
Pandemic, 27 Months (April 2020–June 2022)	54 (66.67)	45 (67.16)	9 (64.28)	0.835
**Demographic characteristics**				
Median Age (IQR)	70 (61–79)	69 (59–79)	70 (64–77)	0.685
Female Gender	48 (59.3)	39 (58.2)	9 (64.3)	0.674
**Inflammatory markers**				
Median Procalcitonin (IQR)	3.56 (1.08–22.81)	4.00 (1.08–25.13)	1.91 (1.08–4.63)	0.183
Median CRP (IQR)	213.6 (140.9–329.5)	214.3 (150.3–301.6)	174.4 (98.3–364.0)	0.861
**Clinical characteristics**				
Mechanical ventilation	77 (95.06)	66 (98.51)	11 (78.57)	**0.002**
COVID-19	38 (46.91)	34 (50.75)	4 (28.57)	0.223
Fever > 38 °C	47 (58.02)	37 (55.22)	10 (71.43)	0.264
Comorbidity	66 (81.48)	54 (80.60)	12 (85.71)	0.654
Median Apache Score (IQR)	20 (15–27)	19.5 (15–24)	27 (20–30)	0.204
Malignancy	36 (44.44)	30 (44.78)	6 (42.86)	0.895
Healthcare associated	51 (62.96)	40 (59.70)	11 (78.57)	0.184
**Infection site**				
Tracheal aspirate	69 (85.18)	61 (91.04)	8 (57.14)	**0.001**
Blood	24 (29.63)	17 (25.37)	7 (50.00)	0.066
Urine	1 (1.23)	1 (1.49)	0	N/A
**Antimicrobial resistance**				
Amikacin resistance	65 (80.25)	55 (82.09)	10 (71.43)	0.362
Gentamicin resistance	65 (80.25)	55 (82.09)	10 (71.43)	0.362
Meropenem resistance	73 (90.12)	62 (83.78)	11 (78.57)	0.111
Imipenem resistance	73 (90.12)	62 (83.78)	11 (78.57)	0.111
Ceftazidime resistance	74 (91.36)	62 (83.78)	12 (85.71)	0.409
Piperacillin/Tazobactam resistance	74 (91.36)	62 (83.78)	12 (85.71)	0.409
Ciprofloxacin resistance	73 (90.12)	62 (83.78)	11 (78.57)	0.067
Colistin resistance	0	0	0	N/A

Significant values are in [bold].

The antimicrobial resistance rates of 81 isolates were found to be 90.12% (73/81) for imipenem, 90.12% (73/81) for meropenem, 91.36% (74/81) for ceftazidime, 80.25% (65/81) for amikacin, 80.25% (65/81) for gentamicin, 91.36% (74/81) for piperacillin-tazobactam, and 90.12% (73/81) for ciprofloxacin. The distribution of MIC50 and MIC90 values of all isolates (n = 81) were shown in Table 3. All isolates were susceptible to colistin and 90.12% (73/81) of isolates were concluded to be MDR. Out of 27 isolates that PFGE was performed on, all isolates were resistant to ceftazidime, 26 were resistant to amikacin, and 25 were resistant to gentamicin.

**Table 3:** MIC50 and MIC90 distributions of all isolates with resistance characteristics.

Antimicrobial	Range	MIC50	MIC90	Susceptible (%)	Intermediate (%)	Resistant (%)
Amikacin	≤ 2 to ≥ 64	≥ 64	≥ 64	16 (19.75)	0.00	65 (80.25)
Gentamicin	≤ 1 to ≥ 16	≥ 16	≥ 16	16 (19.75)	0.00	65 (80.25)
Meropenem	≤ 0.25 to ≥ 16	≥ 16	≥ 16	8 (9.88)	0.00	73 (90.12)
Imipenem	≤ 0.25 to ≥ 16	≥ 16	≥ 16	8 (9.88)	0.00	73 (90.12)
Ceftazidime	2 to ≥ 64	≥ 64	≥ 64	7 (8.64)	0.00	74 (91.36)
Ciprofloxacin	≤ 0.25 to ≥ 4	≥ 4	≥ 4	3 (3.70)	5 (6.17)	73 (90.12)
Colistin	≤ 0.5 to 1	≤ 0.5	≤ 0.5	81 (100.00)	0.00	0.00
Piperacillin Tazobactam	≤ 4 to ≥ 128	≥ 128	≥ 128	7 (8.64)	0.00	74 (91.36)

Weekly feedback and education on hand hygiene measures have enhanced hand hygiene compliance scores from 70.0 to 97.5%. No growth of *A. baumannii* has been detected since February 2022. As of July 2022, containment has been achieved for five months.

**Figure 1:**
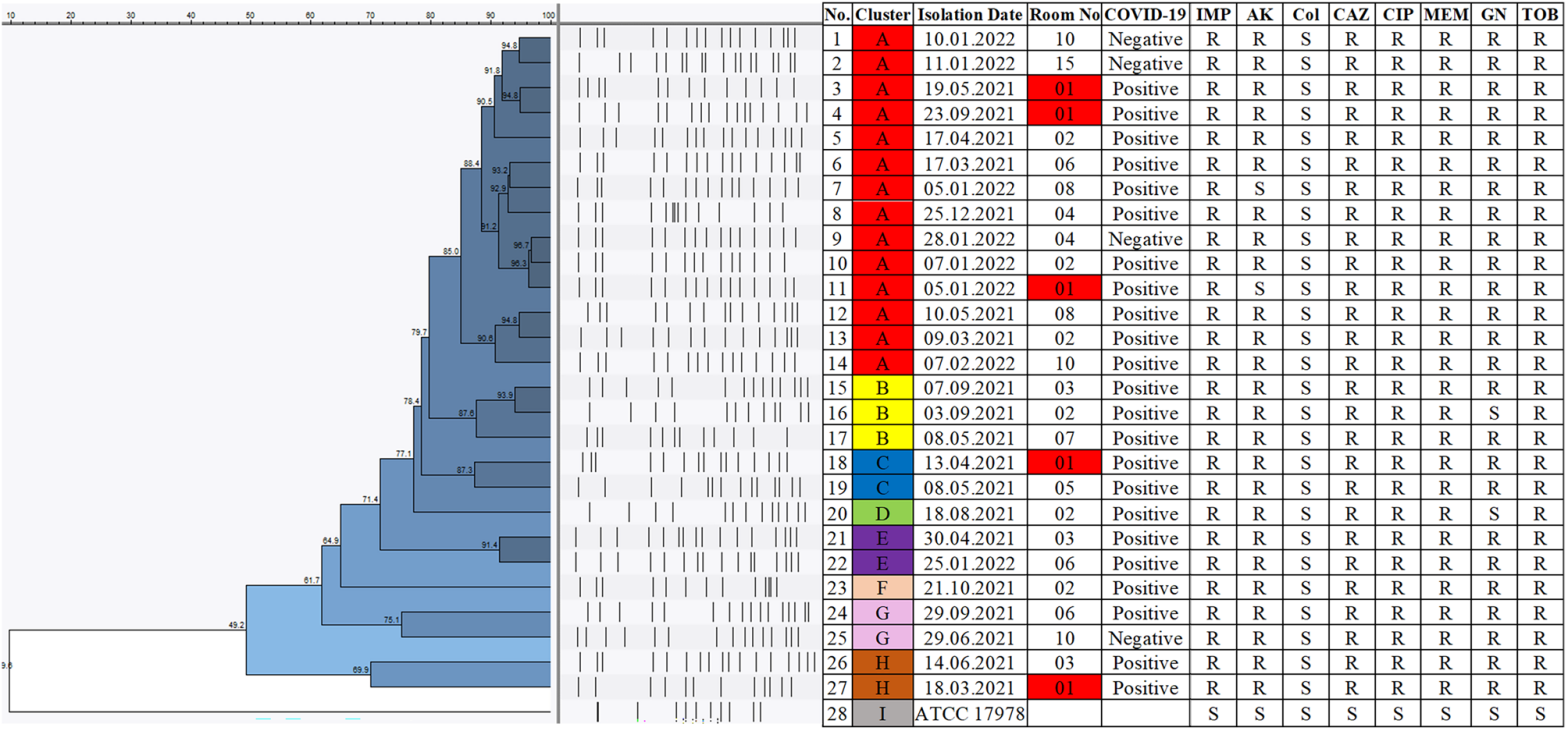
Cluster analysis of *A. baumannii* samples using PFGE results. Each color represents a different cluster of isolates. IMP, imipenem; AK, amikacin; Col, colistin; CAZ, ceftazidime; CIP, ciprofloxacin; MEM, meropenem; GN, gentamicin; TOB, tobramycin; R, resistant; S, susceptible.

Clusters of A, B, C, D, E, F, G, and H were identified from 27 MDR *A. baumannii i*solates (Fig. 1). Isolates with a similarity score of 85% and above were considered to belong to the same cluster. PFGE cluster A was observed to be the dominant cluster with 14 members isolated from 14 different individuals. *A. baumannii* ATCC 17978 was used as the control strain and its genetic relatedness to outbreak isolates was found to be 9.6%.

## Discussion

MDR *A. baumannii* infections have risen dramatically during the COVID-19 pandemic and have become a major concern in ICUs^13,26^. In our institution, healthcare-associated *A. baumannii* infections have increased during the pandemic period (0.97% vs. 1.83%, *p* < 0.01), highest in 2021 (2.28%), however, no statistically significant increase was observed in case fatality rate (83.3% vs 81.5%, *p* = 0.835).

Application of IPC measures during the COVID-19 pandemic induced a decrease of MDR organisms^27^, however, incorrect use of PPE, lack of training in cleaning and disinfection, high turnover of the janitorial staff, and work overload may have contrasted this outcome in this hospital. It has been noticed that the use of double gloves by healthcare personnel when treating COVID-19 patients has a detrimental impact on compliance to hand hygiene. Additionally, compliance with blinded cleaning by newly recruited janitorial staff was noted to be inadequate which has raised our concern for environmental contamination.

Environmental contamination has a critical role in the spread of nosocomial infections^28,29^. A different study has shown that screening surroundings and medical devices for possible sources during outbreaks were beneficial^30^. In our study, environmental sampling was performed for surveillance because of an increase in *A. baumannii* infections with similar susceptibility patterns to existing antibiotics. Detection of *A. baumannii* on aspiration jars, IV pumps, bed control panels, and stethoscopes has pointed out the need for further surveillance and investigation of specimens through their clonal relatedness. Hence, PFGE was applied to isolates obtained from patients during the increased rate of infections from March 2021 to February 2022. Cluster analysis of 27 isolates (Fig. 1) resulted in eight different clones. Isolates with clonality scores equal to and over 85% were considered clonal. Most isolates belonged to Cluster A (14/27) and B (3/27). Six out of eight clusters had isolates coming from rooms number 1 and 2, exhibiting the likelihood of cross-contamination. Additionally, four out of 27 patients did not have the SARS-CoV-2 infection. Three of the non-COVID-19 patients belonged to Cluster A and the remaining isolate belonged to Cluster G, hence, we could not exhibit any association between the presence of COVID-19 and *A*, *baumannii* clusters.

Eventually, isolation dates of samples belonging to cluster A were found to cover the dates from March 2021 to February 2022, which concluded an *A. baumannii* outbreak at the ICU followed by immediate actions taken regarding infection control measures. The major limitation of this study was not being able to apply the PFGE method on environmental specimens and earlier patient isolates due to stocked bacteria samples exhibiting no growth after inoculation. PFGE method was decided to be performed after the suspicion of an outbreak, by the time older samples were requested back from stocks, the pre-pandemic and pre-outbreak samples were not viable. The study was not able to address the epidemic clones of *A. baumannii* isolates as MLST and WGS was not performed, however, PFGE exhibited adequate discriminatory power on assessing clonal relatedness of samples isolated from the same hospital^16,17^. The isolates resistant to more than 3 classes of antibiotics were concluded to be MDR however, as antibiotic susceptibility testing of 9 different antimicrobial categories was not performed, the study was unable to assess potential XDR^31^.

Regarding the dissemination of nosocomial pathogens, previous studies have shown that an insufficient number of healthcare workers, failure to comply with PPE, lack of education, miscommunication, and violations of environmental cleanliness facilitate the spread of resistant microorganisms^10,32^. *A. baumannii* infection elimination program helped us manage the epidemic holistically. In a recent study based on the permanent elimination of *A. baumannii* infection spreading within ICU during COVID-19, the components of the infection control program (such as hand hygiene, correct use of PPE, contact precautions, environmental cleaning and disinfection procedure, antimicrobial management) were proven to be effective^33^. Additionally, in this study, it was shown that education, communication, follow-up, and multidisciplinary approach are crucial in the management of MDR *A. baumannii* infection.

## Conclusion

This report shows that MDR *A. baumannii* infection occurs more frequently during the COVID-19 pandemic compared to the pre-pandemic era. Existing infection control measures and their application are vital in sustaining appropriate working conditions. Co-infection of the lungs by SARS-CoV-2 and *A. baumannii* increases fatality. Eventually, our findings illuminate that the *A. baumannii* infection elimination program has contributed to the containment of this outbreak.

## Supplementary Information

Supplementary Information 1.

Supplementary Information 2.

## Data Availability

All data used and analyzed in this study are available in the Supplementary Information files section.
