# Supplementary material for: The association between Acinetobacter baumannii infections and the COVID-19 pandemic in an intensive care unit
Source: Sci Rep. 2022 Dec 2;12:20808. doi: 10.1038/s41598-022-25493-8 (PMC9716169; doi:10.1038/s41598-022-25493-8)
Supplement: Supplementary file 1 — Supplementary Information 1. [file 41598_2022_25493_MOESM1_ESM.pdf]

Supplementary data 1- Pulsed-Field Gel Electrophoresis (PFGE) experiment results with uncropped gel images.

Ethidium bromide was used for gel staining and visualization of the results was done using ChemiDoc™ XRS+ System with Image Lab™ Software (BIO-RAD, USA).The isolates labelled as ‘X’ belongs to either another study group, was discarded due to poor quality for analysis or was test samples for repeatability. As a single run only can be used for testing 13 isolates there were more than 1 run for this experiment for a total of 28 isolates. Repeatability of the experiment was tested by running same isolates on different PFGE runs.

Control ATCC X 24 23 9 7 11 10 17 8 27 26 X Control

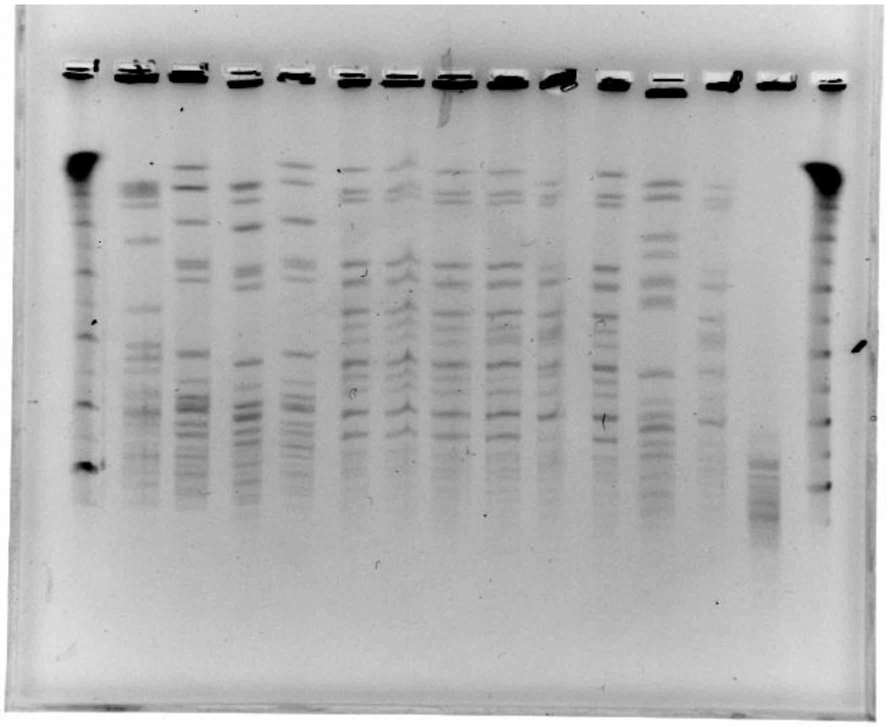

Supplementary Figure 1 Uncropped gel with Control, ATCC, X, isolate 24, 23, 9, 7, 11, 10, 17, 8, 27, 26, X, Control results respectively.

Control 6 5 21 22 18 19 25 3 20 4 15 16 X Control

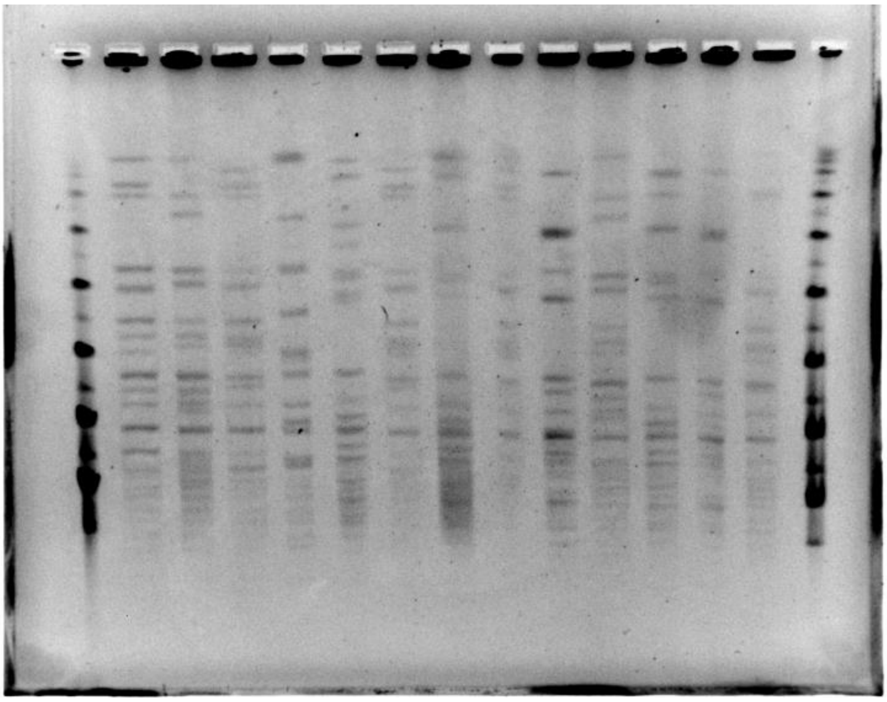

Supplementary Figure 2. Illustration of the uncropped gel with Control, isolates 6, 5, 21, 22, 18, 19, 25, 3, 20, 4, 15, 16, X and Control results respectively.

Ctrl 1 14 2 12 13 X X X X X Control

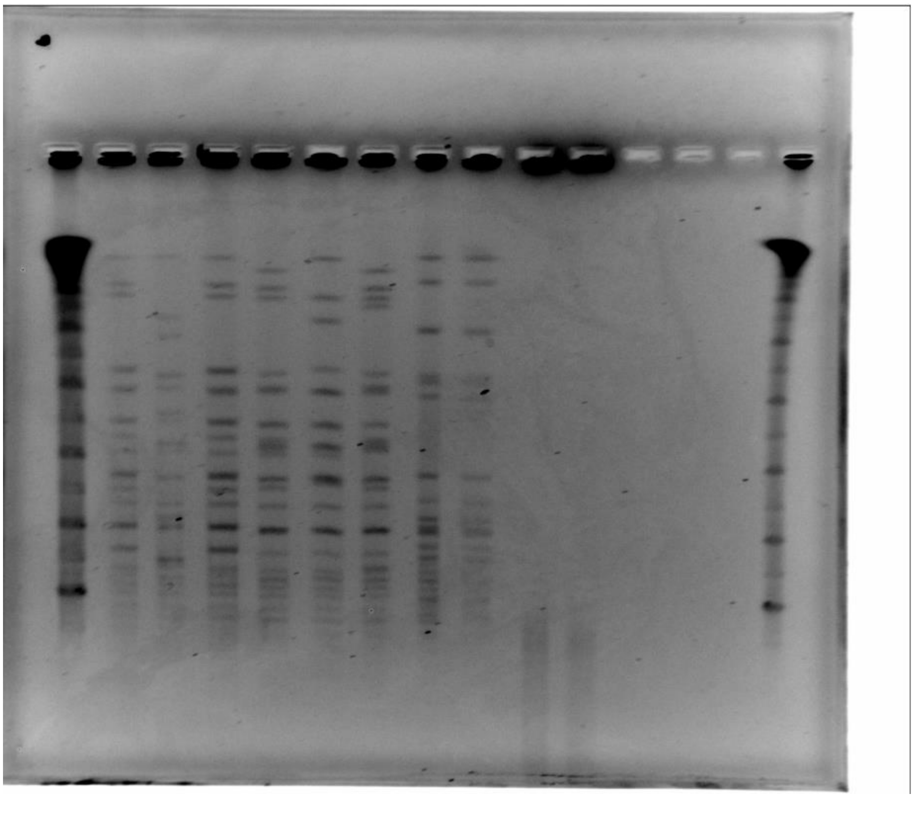

Supplementary Figure 3. Illustration of the uncropped gel with Control, isolates 1, 14, 2, 12, 13, X, X, X, X, X, Blank, Blank, Blank, Control, respectively

Repeatability test for the PFGE protocol on different runs

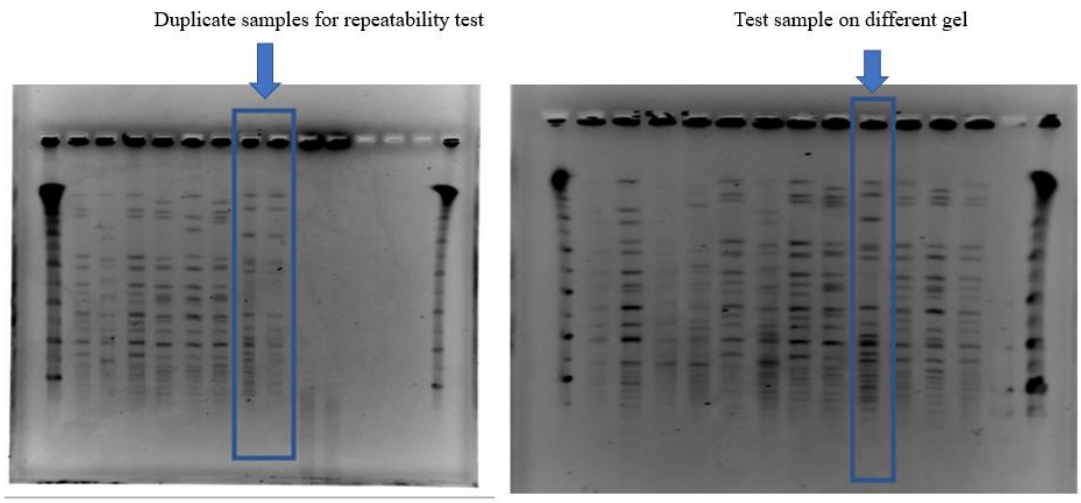

Supplementary Figure 4. Illustration of the same isolate repeated on same PFGE run (in duplicate) and a different run respectively, for the assurance of the repeatability of the protocol.
